# Supplementary material for: Effect of Hypoxia on Glucose Transporter 1 and 3 Gene Expression in Placental Mesenchymal Stem Cells Derived from Growth-Restricted Fetuses
Source: Genes (Basel). 2022 Apr 25;13(5):752. doi: 10.3390/genes13050752 (PMC9140667; doi:10.3390/genes13050752)
Supplement: Supplementary file 1 [file genes-13-00752-s001.zip › genes-1681827-supplementary.pdf]

supplementary file 1: Flow cytometry data of placenta mesenchymal stem cells

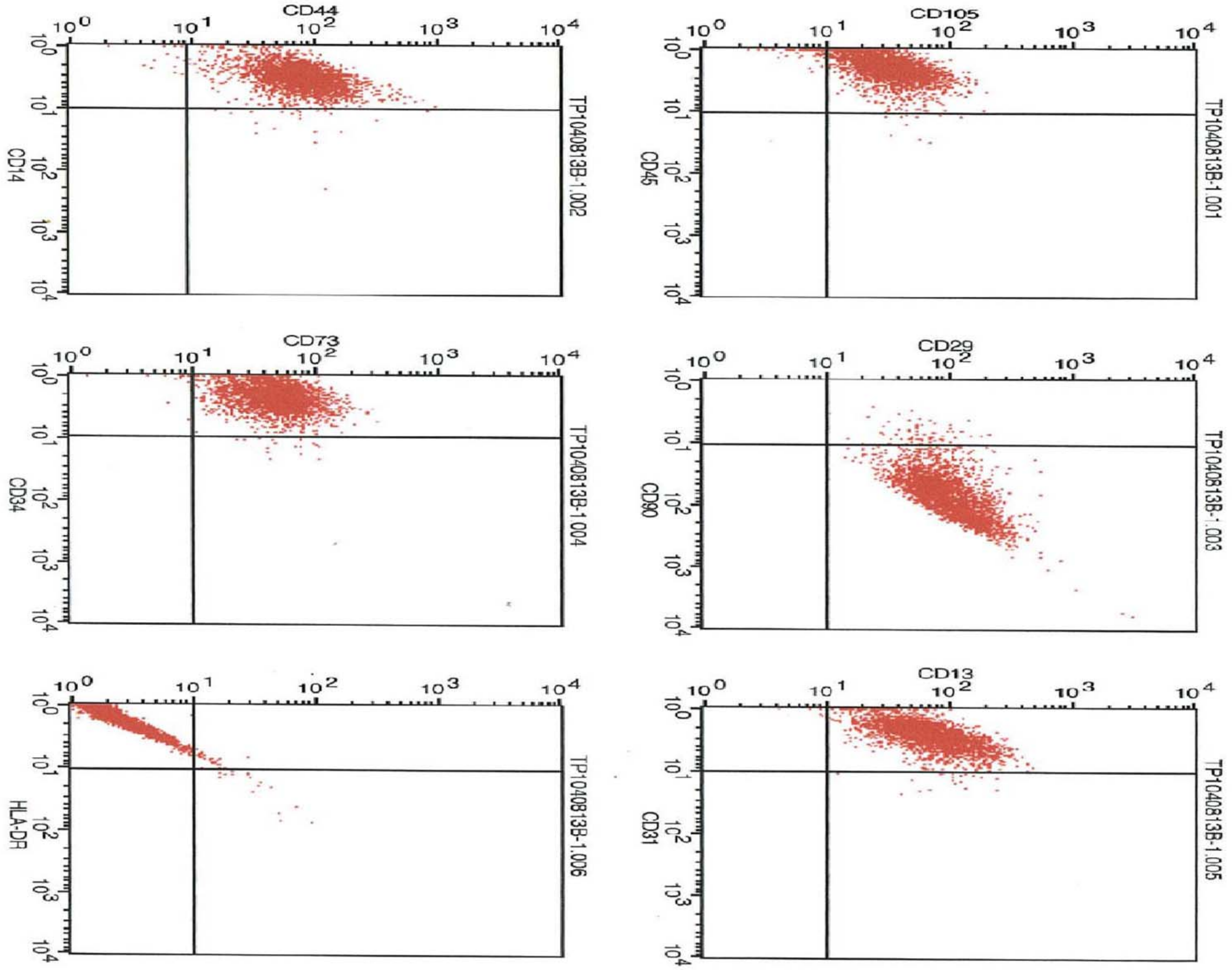

Supplementary file 2

Western blot of HIF-1α

The PMSCs were lysed in RIPA buffer (Millipore, MA, USA) by using proteinase inhibitors (Millipore, MA, USA). After centrifugation at 12,000 rpm for 15 min at 4 °C, the protein concentration was determined using the Bradford protein assay (Biorad La-boratories, CA, USA). The lysates were subjected to sodium dodecyl sulfate–polyacrylamide gel electrophoresis (SDS–PAGE), and the separated proteins were sub-sequently transferred onto polyvinylidene fluoride membranes (Millipore, MA, USA). The antibodies for HIF-1 used: (GTX628480, 1:1000, Genetex, Hsinchu, Taiwan). Horseradish peroxidase–conjugated antibodies and chemiluminescence reagents were obtained from Millipore. The signal intensity of autoradiograms was quantified using VisionWorks (UVP, Analytik Jena GmbH, Germany) after normalization to the corre-sponding actin intensity.

Case 1

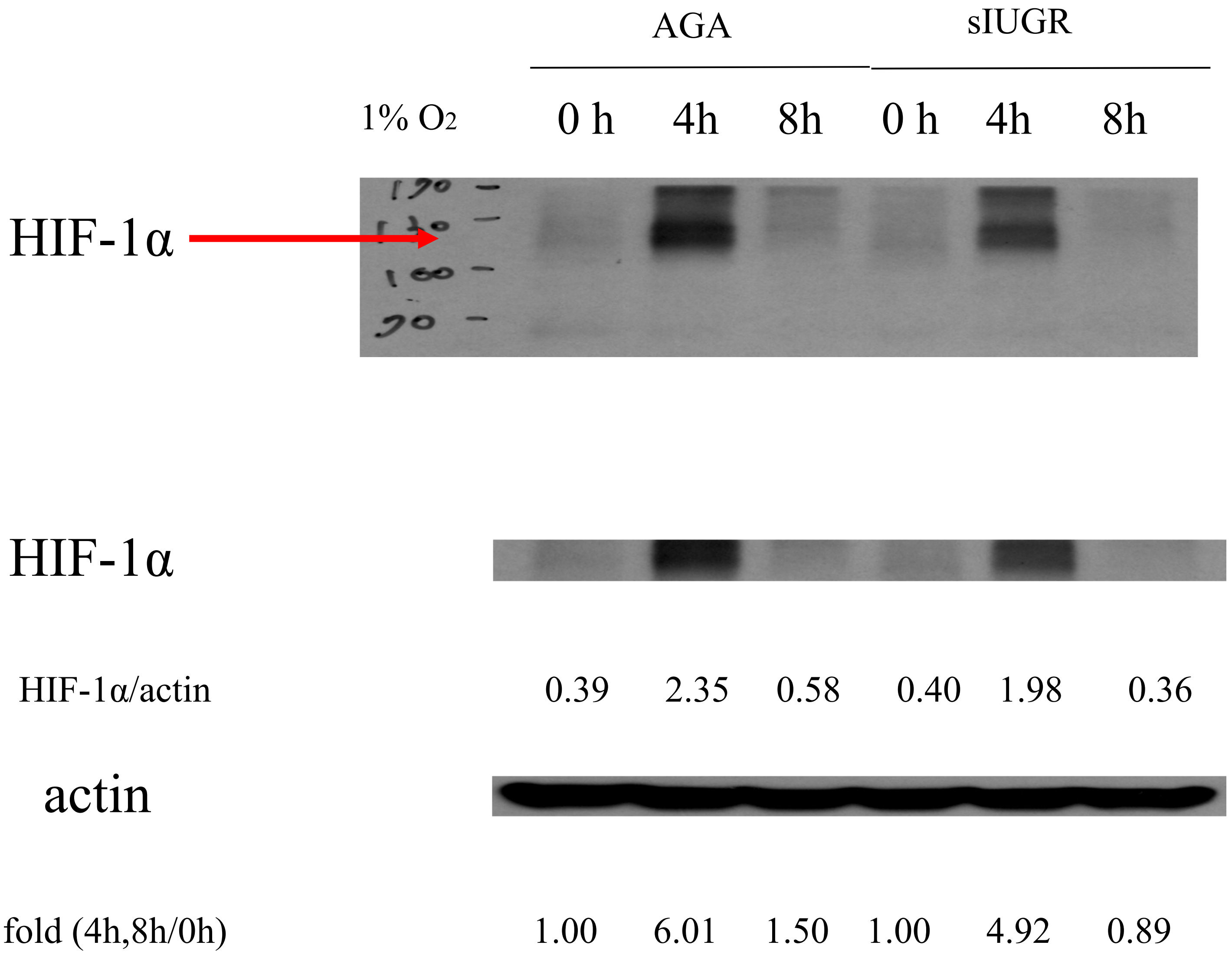
AGA
sIUGR
1% O2
0 h 4h 8h 0 h 4h 8h
HIF-1α
HIF-1α
HIF-1α/actin 0.39 2.35 0.58 0.40 1.98 0.36
actin
fold (4h,8h/0h) 1.00 6.01 1.50 1.00 4.92 0.89

Case 2

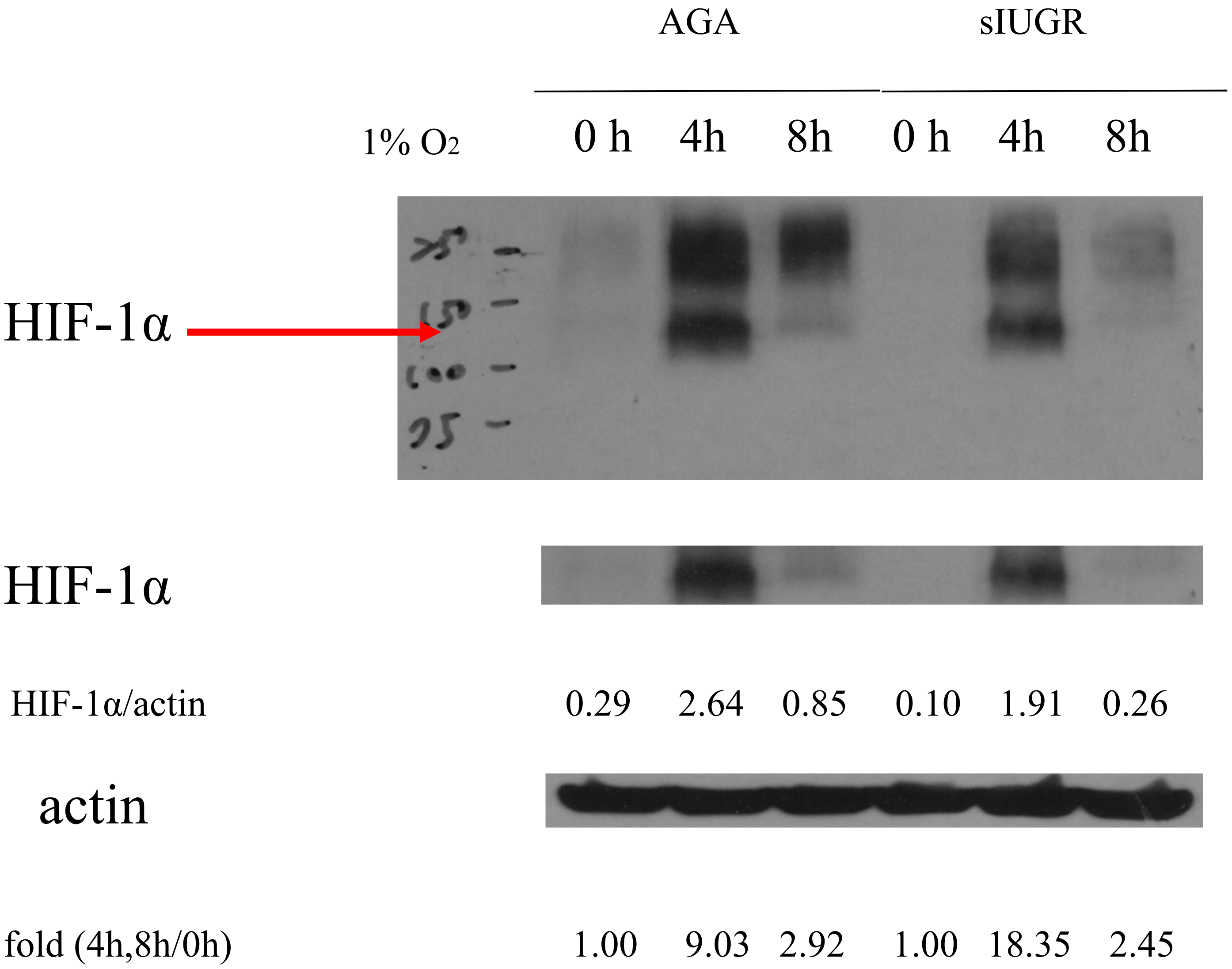
AGA
sIUGR
1% O_2
0 h 4h 8h 0 h 4h 8h
HIF-1α
HIF-1α
HIF-1α/actin 0.29 2.64 0.85 0.10 1.91 0.26
actin
fold (4h,8h/0h) 1.00 9.03 2.92 1.00 18.35 2.45

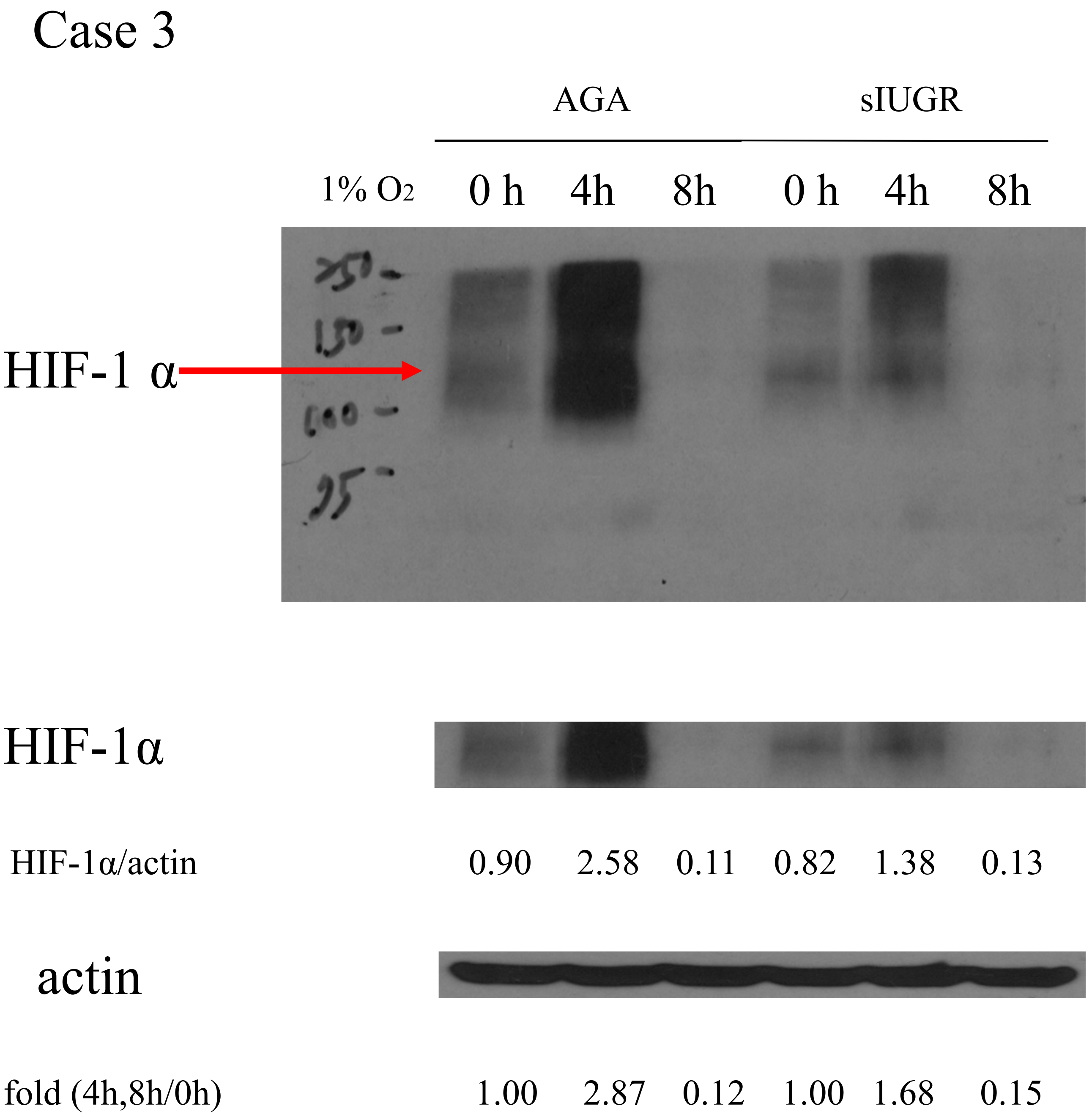

Case 3
AGA
sIUGR
1% O2
0 h
4h
8h
0 h
4h
8h
HIF-1 α
HIF-1α
HIF-1α/actin
0.90
2.58
0.11
0.82
1.38
0.13
actin
fold (4h,8h/0h)
1.00
2.87
0.12
1.00
1.68
0.15

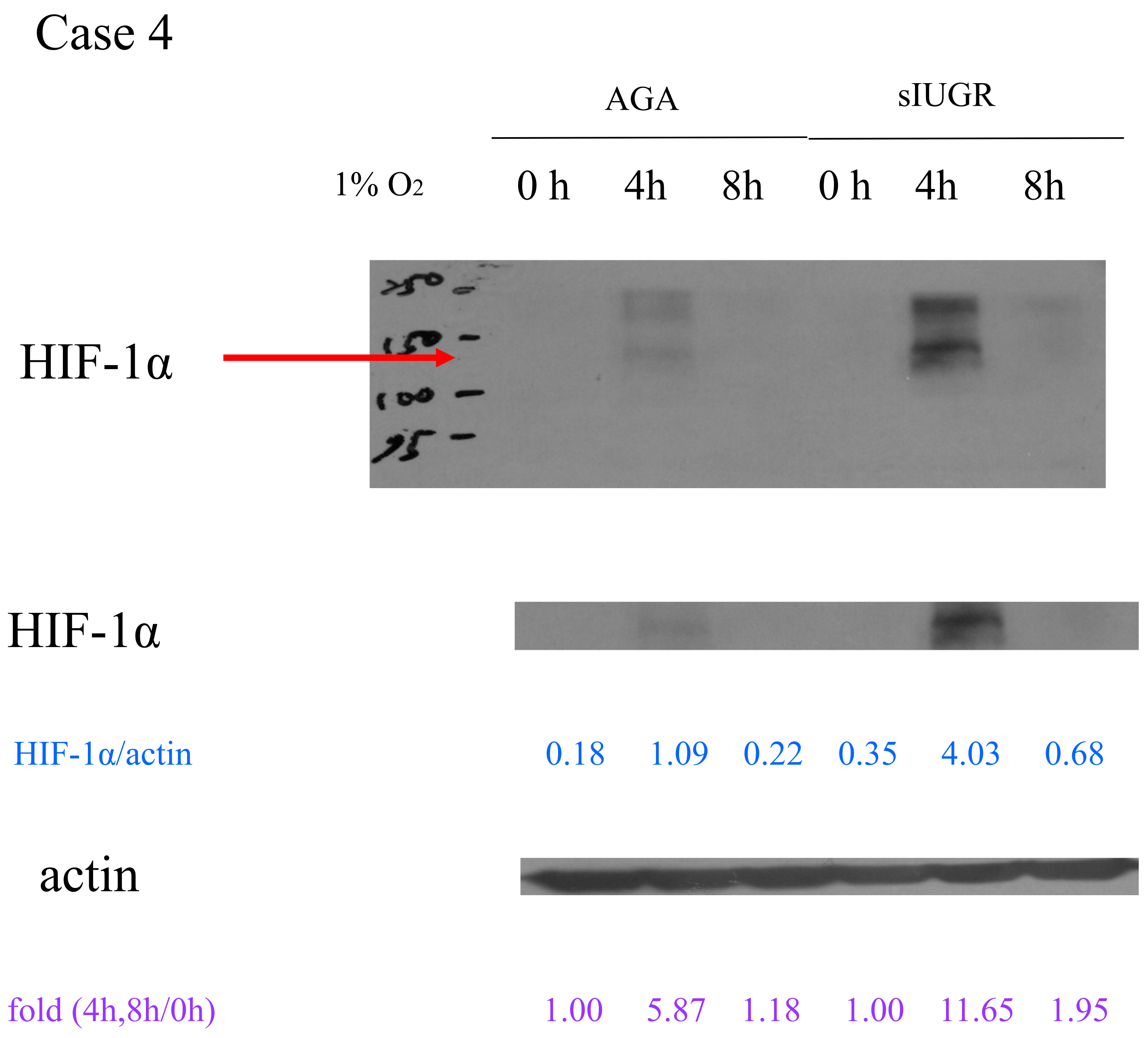
Case 4
AGA
sIUGR
1% O2
0 h
4h
8h
0 h
4h
8h
HIF-1α
HIF-1α
HIF-1α/actin
0.18
1.09
0.22
0.35
4.03
0.68
actin
fold (4h,8h/0h)
1.00
5.87
1.18
1.00
11.65
1.95

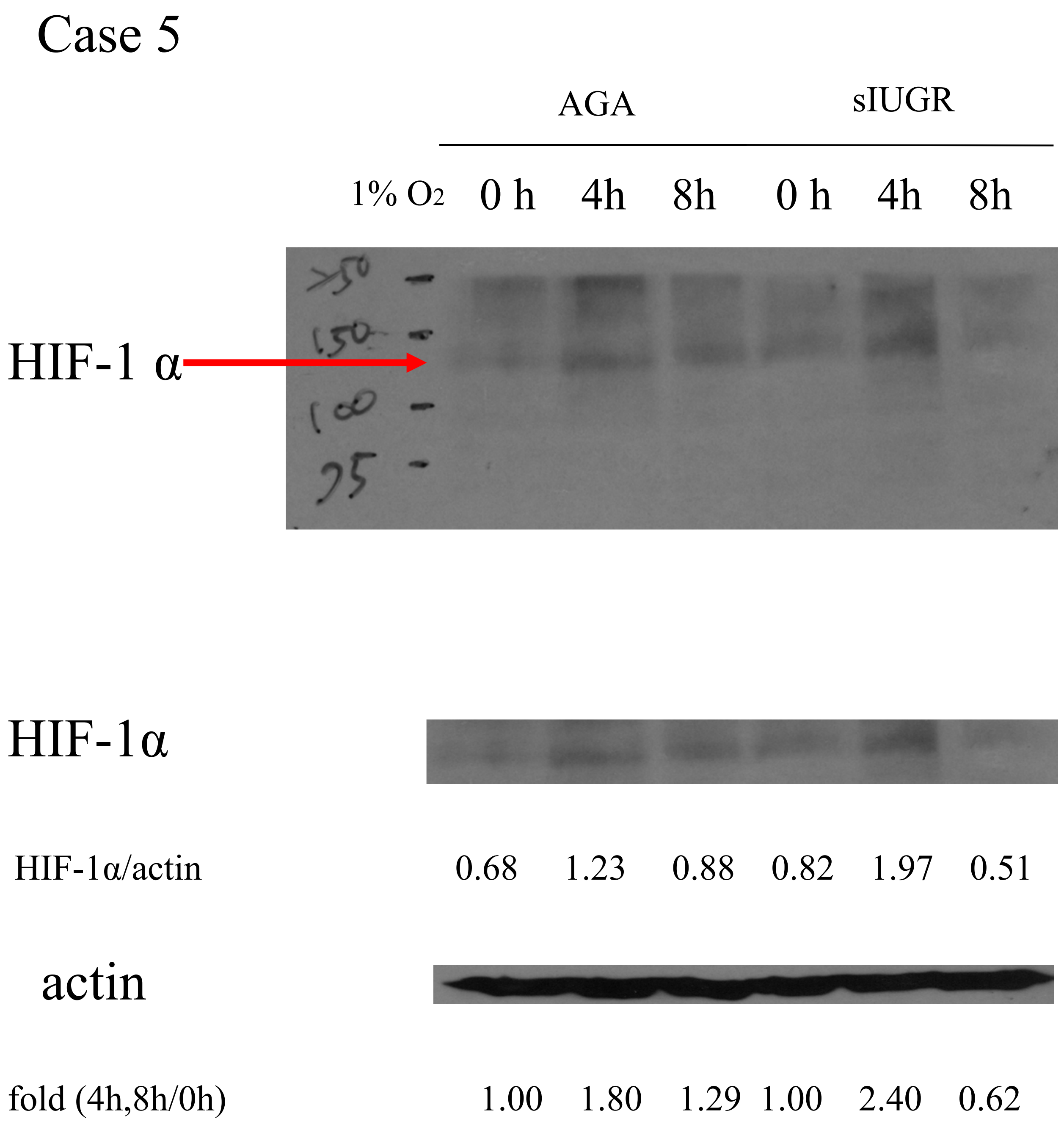
Case 5
AGA
sIUGR
1% O2
0 h 4h 8h 0 h 4h 8h
HIF-1 α
HIF-1α
HIF-1α/actin 0.68 1.23 0.88 0.82 1.97 0.51
actin
fold (4h,8h/0h) 1.00 1.80 1.29 1.00 2.40 0.62

Case 6

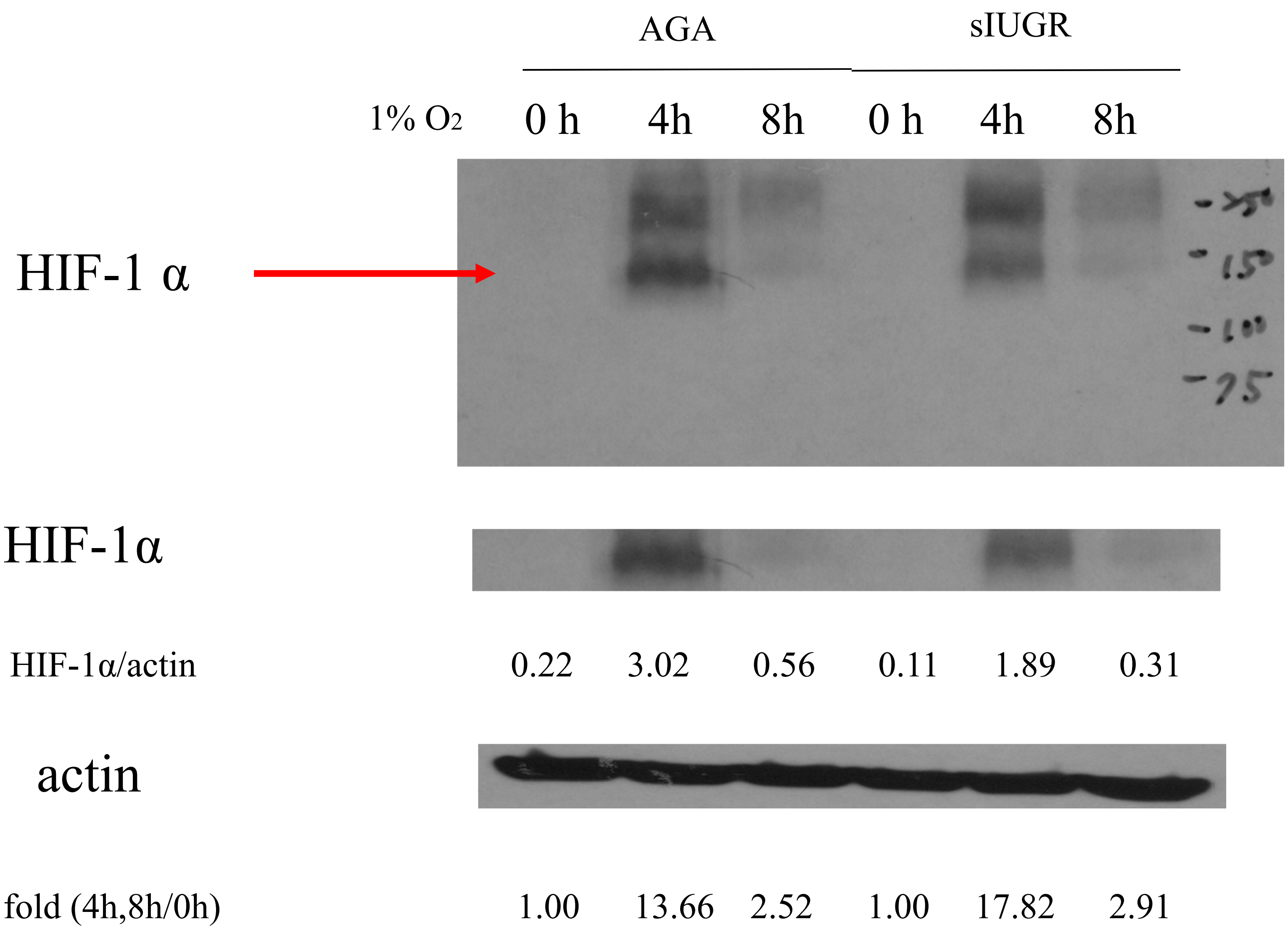
AGA
sIUGR
1% O2
0 h
4h
8h
0 h
4h
8h
HIF-1 α
250
150
100
75
HIF-1α
HIF-1α/actin
0.22
3.02
0.56
0.11
1.89
0.31
actin
fold (4h,8h/0h)
1.00
13.66
2.52
1.00
17.82
2.91
